# Investigation of the Relationship between Plasma Nesfatin-1 Levels and Neutering in Dogs

**DOI:** 10.3390/ani14192854

**Published:** 2024-10-03

**Authors:** Gokcen Guvenc-Bayram, Zeynep Semen, Murat Yalcin

**Affiliations:** 1 Department of Physiology, Faculty of Veterinary Medicine, Dokuz Eylul University, Izmir 35890, Turkey; 2 Department of Biochemistry, Faculty of Veterinary Medicine, Dokuz Eylul University, Izmir 35890, Turkey; zeynep.semen@deu.edu.tr; 3 Department of Physiology, Faculty of Veterinary Medicine, Bursa Uludag University, Bursa 16059, Turkey; muraty@uludag.edu.tr

**Keywords:** dog, dopamine, female and male, nesfatin-1, neutering, serotonin, T4, TSH

## Abstract

**Simple Summary:**

Neutering, which includes orchiectomy for males and ovariohysterectomy for females, is a common practice in dogs to control populations and prevent health issues. Despite its benefits, neutering can lead to hormonal changes that may contribute to a tendency toward obesity over time. Our study investigated how neutering in both female and male dogs affects hormones involved in metabolism and appetite regulation, specifically nesfatin-1, serotonin, dopamine, TSH, and T4. We observed that neutering decreased levels of nesfatin-1, serotonin, and T4, and increased TSH levels in both genders. These hormonal changes could be part of a mechanism contributing to the higher risk of obesity observed in neutered dogs in the long term.

**Abstract:**

Neutering of dogs, whether male or female, provides various benefits such as contraception, population control, and the prevention of reproductive disorders and undesirable sexual behaviors. However, it is also associated with an increased risk of obesity, which may be directly linked to post-neutering hormonal changes. Our study aims to determine the effects of neutering on plasma levels of nesfatin-1, serotonin, dopamine, TSH, and T4—hormones implicated in obesity and metabolic regulation. Fourteen dogs (seven males and seven females), aged between 1 and 3 years, were included in this study. Male dogs underwent orchiectomy and females underwent ovariohysterectomy. Blood samples were collected before surgery and on days 7 and 14 post-operatively to measure the plasma levels of these hormones using ELISA. The results showed a significant decrease in nesfatin-1, serotonin, and T4 levels, along with a significant increase in TSH levels in both male and female dogs post-neutering. While these hormonal changes are likely part of the body’s adaptive response to neutering, they may represent a potential mechanism that contributes to the long-term tendency toward obesity in neutered dogs.

## 1. Introduction

Neutering, encompassing procedures such as orchiectomy for males and ovariohysterectomy or ovariectomy for females, is widely practiced in dogs for its benefits, including contraception, population control, and the prevention of reproductive disorders and undesirable sexual behaviors [1]. However, neutering is also associated with several disadvantages, such as potential anesthesia-related and surgical complications, increased risk of prostate cancer, musculoskeletal and endocrine disorders, and a higher propensity for obesity [2]. Obesity, a prevalent nutritional disorder, is of growing concern among neutered dogs. Factors such as reduced metabolic rate, heightened food intake, and decreased activity levels may contribute to obesity following neutering [3].

Nesfatin-1, an 82-amino-acid peptide with anorexigenic effects, is derived from the post-translational processing of the N-terminal fragment of nucleobindin-2 (*NUCB2*) [4]. This peptide is expressed in both the central nervous system nuclei that regulate food intake and peripheral tissues and is capable of traversing the blood–brain barrier bidirectionally [5]. Notably, *NUCB2*/nesfatin-1 is present in reproductive organs, suggesting its involvement in reproductive processes. In rodents, *NUCB2*/nesfatin-1 mRNA expression is prominent in the ovaries, uterus, placenta, testes, and epididymis [6]. Additionally, nesfatin-1 has garnered attention for its role in the regulation of food intake and its contribution to the physiopathology of obesity [7,8,9].

Serotonin, a neurotransmitter primarily known for its role in mood regulation and well-being, is also crucial for appetite control. It acts on specific receptors in the hypothalamus to modulate feeding behavior and satiety signals [10]. Reduced serotonin levels have been associated with increased food intake and a higher risk of obesity [11]. The presence of serotonin receptors in reproductive organs, such as the ovaries, uterus, placenta, testes, and epididymis, suggests a potential role in reproductive processes [12].

Dopamine, another key neurotransmitter, is involved in the brain’s reward and pleasure pathways, motivation, and movement control [13]. It influences food reward processing and reinforcement, affecting food-seeking behaviors and appetite regulation [14]. Dysregulation of dopamine signaling has been implicated in obesity and related metabolic disorders [15]. Additionally, dopamine receptors are present in various reproductive organs, including the ovaries, uterus, testes, and epididymis, indicating their involvement in reproductive functions [16].

Thyroid-stimulating hormone (TSH) and thyroxine (T4) are critical for thyroid function and metabolic regulation. TSH stimulates the thyroid gland to produce T4, which is subsequently converted to the active form, triiodothyronine (T3), in peripheral tissues [17]. Thyroid hormones are essential for regulating metabolic rate, energy expenditure, and body weight [18]. Altered thyroid function is associated with changes in body composition, including increased adiposity [19]. Furthermore, thyroid hormones interact closely with the hypothalamic–pituitary–gonadal axis [20].

In light of this information, our study aims to evaluate the post-neutering levels of nesfatin-1, serotonin, dopamine, and thyroid hormones.

## 2. Materials and Methods

### 2.1. Animals and Experimental Protocols

This study included a total of 14 mixed-breed dogs (7 females and 7 males), aged between 1 and 3 years, which were brought to the Temporary Animal Shelter of the Manisa Metropolitan Municipality for routine neutering procedures. The dogs were individually housed in standard cages with ad libitum access to food and water. To ensure the inclusion of only healthy animals in the study, each dog underwent a comprehensive clinical evaluation before the study. This included a thorough physical examination conducted by a licensed veterinarian, assessing vital signs and screening for any signs of infectious diseases or chronic health conditions. In addition to the clinical evaluation, all dogs underwent hematological and serum biochemistry analyses to further exclude any underlying health issues. Blood tests included a complete blood count and a comprehensive serum biochemistry panel, which evaluated liver and kidney function, electrolyte balance, and inflammatory markers. Only clinically healthy dogs with normal results were selected for the study.

Following the health screening, all dogs were acclimatized to the research environment in an isolated ward at the center for a minimum of 24 h before surgery. This acclimatization period was essential to reduce stress and ensure stable physiological base-lines, thus avoiding confounding variables related to changes in the external environment.

For the surgical procedures, all dogs were subjected to a standardized anesthetic protocol to minimize variability. Analgesia was administered with 0.1 mg/kg intramuscular karprofen (5% *w*/*v* (50 mg/mL, Rimadyl^®^ Pfizer, Peapack, NJ, USA), followed by intravenous sedation with 0.2 mg/kg midazolam (Richmond Vet Pharma, Grand Bourg, Bs As, Argentina) and 1 mg/kg ketamine (Ketasol 10%, Interhas, Ankara, Türkiye). Anesthesia was induced with 1–5 mg/kg intravenous propofol (Propofol 1% Fresenius, Fresenius Kabi, Eysins, Switzerland) and maintained using isoflurane (100 mL, Forane^®^ Likid, Abbott, IL, USA) at concentrations between 1.5% and 2%. Ovariohysterectomy (OHE) was performed via a ventral midline celiotomy, with the procedure carefully divided into distinct phases to ensure consistency and precision. These phases were documented in detail for each dog to standardize the surgical approach and minimize potential variability that could affect the study outcomes.

Post-operative care included 0.2 mg/kg subcutaneous karprofen for analgesia. The use of this medication was consistent across all subjects, ensuring that pain management was uniformly applied. This careful control of post-operative analgesia was critical in minimizing the potential confounding effects of pain-induced alterations in the hormonal parameters being measured, particularly serotonin, dopamine, TSH, and T4 levels, which are known to be influenced by stress and pain.

To investigate the effects of neutering on plasma concentrations of nesfatin-1, serotonin, dopamine, TSH, and T4, blood samples were collected before the operation and on days 7 and 14 post-operatively. Blood samples were collected using appropriate-sized needles and under proper sterile conditions. A tourniquet was applied to the left forelimb above the elbow joint, and 2 mL of blood was drawn from the cephalic vein using a needle into vacutainer tubes containing EDTA after cleansing the area. The collected blood samples were immediately placed on ice. After centrifugation at 1800 rpm for 20 min at +4 °C, plasma was then separated and aliquoted into four equal volumes, which were subsequently stored at −80 °C until further analysis.

### 2.2. Determination of Plasma Nesfatin-1, Serotonin, Dopamine, TSH, and T4 Levels

Plasma concentrations of nesfatin-1, serotonin, dopamine, TSH, and T4 were determined using ELISA kits obtained from Bioassay Technology Laboratory (BT-Lab, Shanghai, China). The catalog numbers for the kits were as follows: canine nesfatin-1 (Catalog Number: E0141Ca), canine serotonin (Catalog Number: E0263Ca), canine dopamine (Catalog Number: EA0020Ca), canine TSH (Catalog Number: EA0013Ca), and canine total T4 (Catalog Number: EA0012Ca). Measurements were performed according to the manufacturer’s instructions provided with the kits. In brief, 50 μL of plasma sample was added to microplate wells pre-coated with antibodies specific to each analyte. Following an incubation period and subsequent washing steps, biotin-conjugated antibodies were added to each analyte. After another round of incubation and washing, streptavidin-HRP was added to the wells to form an immune complex. Following further incubation and washing to remove unbound enzymes, a chromogenic HRP enzyme substrate solution was applied to the wells, resulting in a color change to blue. A stop solution was added to terminate the HRP enzyme reaction. The absorbance of the plates was measured at 450 nm using a plate reader (Allsheng.AMR-100). The kits provided detection limits of 0.3–90 ng/mL, 10–640 ng/mL, 5–1000 ng/L, 0.05–20 ng/mL, and 2–600 ng/mL for nesfatin-1, serotonin, dopamine, TSH, and T4, respectively.

### 2.3. Statistical Analysis

All values are presented as mean ± standard deviation (SD) with *p* < 0.05 considered as the level of significance. The assumption of normal distribution for each variable according to female and male groups was evaluated according to the Shapiro–Wilk test and the choice of the test statistic to be used in the repeated measures ANOVA model was decided according to the result of Mauchly’s sphericity test.

Statistical analysis was performed using repeated-measures two-way analysis of variance (RM-ANOVA) to evaluate the effects of neutering on hormone levels over time. Post hoc comparisons were conducted using Bonferroni correction to adjust for multiple comparisons.

## 3. Results

**Figure 1:**
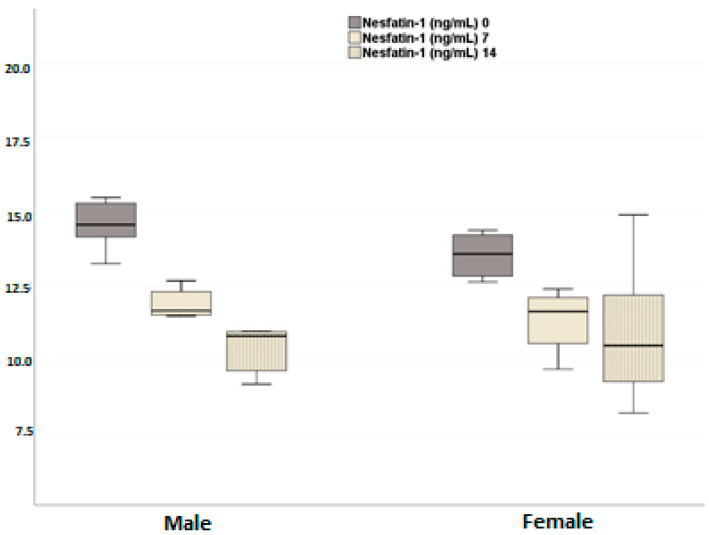
Effects of neutering on plasma nesfatin-1 levels in the female and male dogs. Data are presented as mean ± SD of 7 measurements.

Plasma nesfatin-1 levels significantly decreased in both male and female dogs on days 7 and 14 post-neutering compared to pre-neutering levels (Figure 1) (day 0–day 7, *p* < 0.001; day 0–day 14, *p* < 0.001; day 7–day 14, *p* = 0.001). Although the reduction in nesfatin-1 levels was more pronounced in males compared to females, this difference was not statistically significant (*p* = 0.579). Additionally, the decrease over time did not differ significantly between the male and female groups (*p* = 0.066).

**Figure 2:**
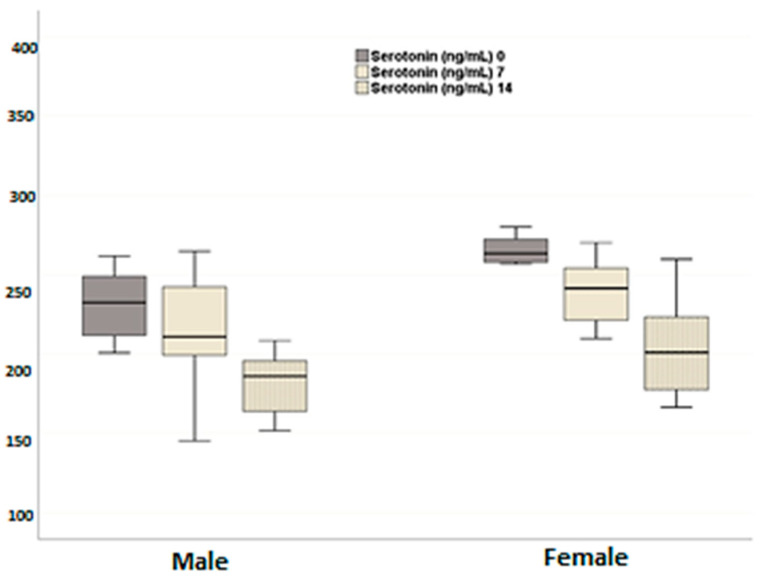
Effects of neutering on plasma serotonin levels in the female and the male dogs. Data are presented as mean ± SD of 7 measurements.

Similarly, plasma serotonin levels were significantly lower in both sexes after neutering compared to pre-neutering levels (Figure 2) (day 0–day 7, *p* = 0.129; day 0–day 14, *p* = 0.002; day 7 × day 14, *p* = 0.006). Interestingly, female dogs exhibited higher plasma serotonin levels than their male counterparts at all time points, although this difference was not statistically significant (*p* = 0.250). Additionally, the decline over time did not differ significantly between the male and female groups (*p* = 0.803).

**Figure 3:**
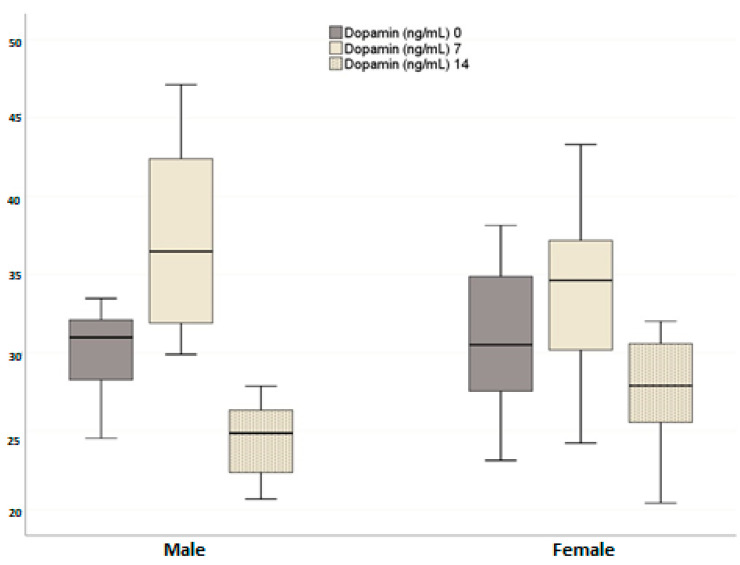
Effects of neutering on plasma dopamine levels in the female and male dogs. Data are presented as mean ± SD of 7 measurements.

In contrast, plasma dopamine levels increased on day 7 after neutering compared to pre-neutering values in both groups but dropped below pre-neutering levels by day 14 (*p* < 0.001). Although no significant difference was found between the male and female groups in terms of plasma dopamine levels (*p* = 0.930), the temporal changes between the groups were significant (*p* = 0.036). In males, while the change from day 0 to day 7 was not statistically significant (*p* = 0.087), the changes between days 0 and 14 (*p* < 0.001) and days 7 and 14 (*p* = 0.006) were statistically significant. In females, the changes at all time points were statistically significant (day 0–day 7, *p* = 0.016; day 0–day 14, *p* = 0.002; day 7–day 14, *p* = 0.016) (Figure 3).

**Figure 4:**
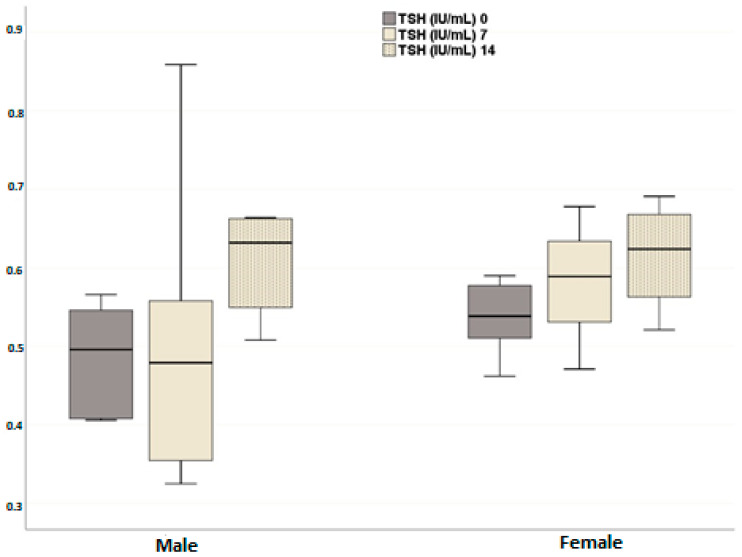
Effects of neutering on plasma TSH levels in the female and male dogs. Data are presented as mean ± SD of 7 measurements.

**Figure 5:**
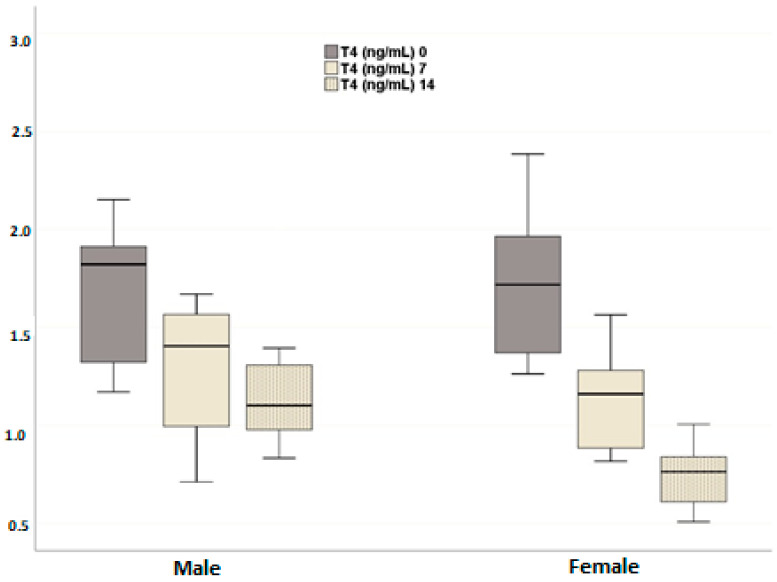
Effects of neutering on plasma T4 levels in the female and male dogs. Data are presented as mean ± SD of 7 measurements.

In both male and female dogs, a significant and consistent increase in plasma TSH levels was observed at all time points after neutering compared to pre-neutering levels (*p* = 0.003) (Figure 4). No significant difference in plasma TSH levels was found between male and female dogs (*p* = 0.199), and the temporal change in plasma TSH levels did not differ between the groups (*p* = 0.259). In contrast, plasma total T4 levels significantly decreased in both sexes after neutering compared to pre-neutering values (*p* < 0.001). While no significant difference in plasma T4 levels was observed between male and female groups (*p* = 0.199), the temporal changes between the groups were significant (*p* = 0.028). In males, the changes between days 0 and 7 (*p* = 0.844) and days 7 and 14 (*p* = 0.394) were not statistically significant, but the total change from day 0 to day 14 was significant (*p* = 0.036). In females, the changes at all time points were statistically significant (day 0 vs. day 7, *p* = 0.006; day 0 vs. day 14, *p* < 0.001; day 7 vs. day 14, *p* = 0.004) (Figure 5). The more pronounced decrease in females suggests a potential sex difference in thyroid hormone regulation following neutering.

## 4. Discussion

Our study is the first to reveal a consistent decrease in plasma nesfatin-1 levels, which are crucial for both reproductive functions and the regulation of nutrition, in the male and female dogs following neutering. Additionally, our findings indicate a significant decrease in plasma serotonin and T4 levels—hormones essential for nutrition, metabolism, and reproductive functions—alongside a significant increase in plasma TSH levels in both sexes.

The most noteworthy finding of our study is the significant decrease in plasma nesfatin-1 levels on the 7th and 14th days post-neutering in both genders, with a more pronounced reduction in the male dogs. Nesfatin-1, a peptide involved in energy homeostasis and appetite regulation, appears to be significantly affected by the removal of gonadal hormones. This finding aligns with previous studies indicating that male dogs may experience more pronounced metabolic and hormonal changes after neutering [6]. Nesfatin-1’s role in the central nervous system, particularly in appetite regulation, underscores its importance in understanding the metabolic consequences of neutering. Nesfatin-1 is known to exert anorexigenic effects, reducing food intake and body weight [4,21]. The observed decline in nesfatin-1 could contribute to increased appetite and subsequent weight gain, a common issue in neutered animals. Obesity development post-neutering is a multifactorial process, and the reduction in nesfatin-1 could be a critical factor. Studies have shown that nesfatin-1 levels are negatively correlated with body mass index (BMI) and body fat percentage, suggesting nesfatin-1’s potential protective role against obesity [22]. The peripheral decrease in nesfatin-1 observed in our study may result from the removal of gonadal organs, which are known to influence nesfatin-1 expression. Gonadal hormones, particularly estrogen and testosterone, have been reported to regulate nesfatin-1 levels, and their removal likely leads to the observed decrease [23]. The decrease in nesfatin-1 following gonadectomy may highlight the peptide’s peripheral role in metabolic regulation. This reduction could predispose neutered dogs to obesity, as decreased nesfatin-1 levels are associated with increased food intake and reduced energy expenditure [24].

Similarly, a significant reduction in plasma serotonin levels post-neutering was observed in both male and female dogs. Notably, female dogs exhibited higher serotonin levels than male dogs pre- and post-neutering. Serotonin, a crucial neurotransmitter involved in mood regulation and behavior, appears to be differentially regulated in male and female dogs, suggesting potential sex-specific protective effects of estrogen on serotonin pathways [25,26,27,28]. These results are consistent with previous findings indicating that estrogen can modulate serotonin levels, potentially mitigating mood disturbances and behavioral changes post-neutering [2]. Moreover, the reduction in serotonin levels following neutering also aligns with observations in human and animal studies, where sex hormone depletion is linked to changes in mood and behavior [29]. Additionally, since serotonin regulates satiety and metabolism, decreased serotonin levels are associated with increased appetite and weight gain [30,31]. The combined effect of reduced sex hormones and lower serotonin levels post-neutering may contribute to a predisposition to obesity in neutered dogs, supported by studies showing a higher incidence of obesity in neutered dogs compared to their non-neutered counterparts [1,32,33,34,35,36].

Dopamine levels exhibited a transient increase on the 7th day post-neutering, followed by a decline below baseline levels by the 14th day. This pattern suggests a complex regulatory mechanism in response to neutering, potentially linked to changes in reward processing and appetite control mechanisms. Dopamine, a key neurotransmitter involved in the brain’s reward and pleasure pathways, motivation, and movement control, plays a crucial role in food reward processing and reinforcement, influencing food-seeking behaviors and appetite regulation [13,14]. The initial spike in dopamine may reflect an acute stress response to surgery and its aftermath, while the subsequent decline could indicate a longer-term adaptation of the reward pathways. Dysregulation of dopamine signaling is implicated in obesity and related metabolic disorders [15]. The mesolimbic dopamine pathway, which mediates the rewarding aspects of food, has been linked to increased food intake and preference for high-calorie foods in states of reduced dopamine activity [14]. The observed changes in dopamine levels suggest that neutering may alter dopamine pathways, potentially contributing to post-neutering weight gain and metabolic changes. Furthermore, dopamine receptors are present in various reproductive organs, including the ovaries, uterus, testes, and epididymis, indicating dopamine’s involvement in reproductive functions [16]. The removal of gonadal hormones during neutering could disrupt the normal dopaminergic regulation of these organs, leading to alterations in dopamine levels observed in our study. This disruption may have downstream effects on behavior and metabolism, further contributing to the risk of obesity in neutered dogs.

A consistent increase in plasma TSH levels post-neutering was observed in both sexes, coupled with a significant decrease in T4 levels. This suggests a disruption in thyroid hormone homeostasis, which could contribute to the metabolic changes observed following neutering. The more pronounced decrease in T4 levels in female dogs indicates potential sex differences in thyroid hormone regulation post-neutering. The hypothalamic–pituitary–thyroid (HPT) axis appears to be responsive to the removal of gonadal hormones, leading to alterations in thyroid function that may influence overall metabolism. Thyroid hormones, specifically T4 and T3, play a crucial role in regulating basal metabolic rate and energy expenditure. A reduction in these hormone levels post-neutering can lead to a decreased metabolic rate, resulting in lower energy expenditure and a predisposition to weight gain. This aligns with the increased risk of obesity observed in neutered dogs, as reported in various studies [37,38]. Plasma thyroid hormone levels have been shown in previous studies to be significantly lower in neutered dogs [39,40]. The thyroid gland contains numerous LH receptors and these are located close to TSH receptors. Continuous stimulation of LH receptors in the thyroid gland may disrupt thyroid function by affecting the normal function of adjacent TSH receptors, contributing to hypothyroidism [41]. These data are compatible with the findings regarding TSH and T4 hormone levels obtained in our study and support the results of our study.

A limitation of our study is the short observation period due to the shelter environment in which it was conducted. As the dogs were rehomed shortly after their stay in the shelter, we were unable to perform long-term follow-up. Monitoring the levels of nesfatin-1, serotonin, dopamine, TSH, and T4 over a longer period post-neutering could have provided more conclusive insights into the potential contribution of these hormones to obesity development. However, our current study serves as a foundation for future long-term research on this topic.

## 5. Conclusions

In conclusion, the findings from the current study indicate that nesfatin-1, serotonin, dopamine, and thyroid hormones undergo significant changes in dogs of both sexes following neutering. While these hormonal alterations are not directly linked to obesity development in the short term, they suggest potential mechanisms that may contribute to metabolic changes post-neutering. Notably, this study introduces new insights into the role of nesfatin-1 in the post-neuter period, particularly its involvement in metabolic and physiological regulation. These results may provide a foundation for future studies investigating the long-term effects of these hormonal changes on obesity and metabolic health in neutered dogs.

## Data Availability

The raw data supporting the conclusions of this article will be made available by the authors on request.
